# Supplementary material for: Identification of Genes Associated with Resistance to Persulcatusin, a Tick Defensin from Ixodes persulcatus
Source: Microorganisms. 2024 Feb 19;12(2):412. doi: 10.3390/microorganisms12020412 (PMC10892762; doi:10.3390/microorganisms12020412)
Supplement: Supplementary file 1 [file microorganisms-12-00412-s001.zip › Supplementary Figure.pdf]

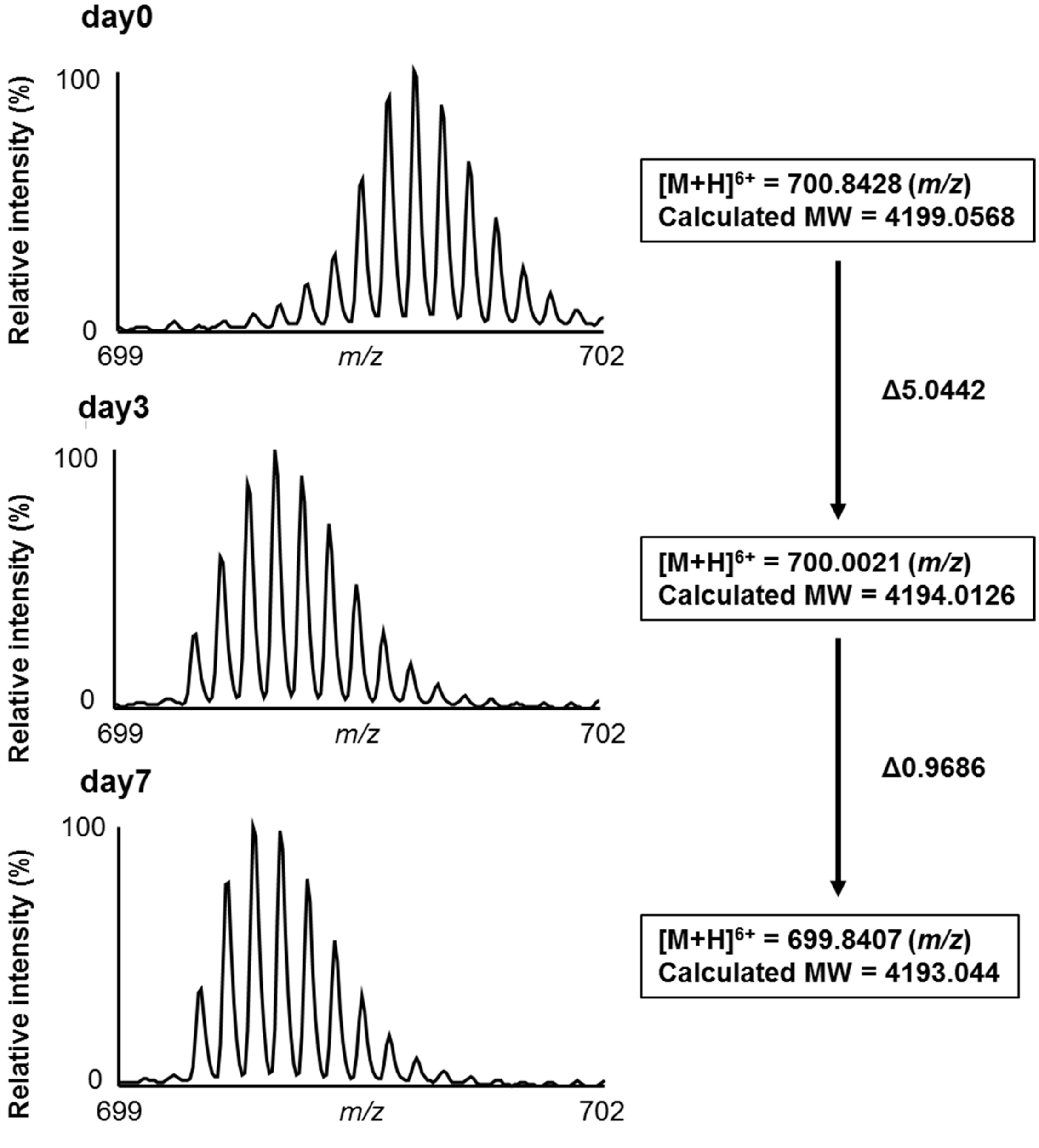

Supplementary Figure S1. Mass spectrometry (MS) analysis of synthesized persulcatusin during aerial oxidation. Mass spectrums (m/z) of protonated molecular [M+H$^+$] caused by electrospay ionization (ESI) were 700.8428, 700.1745, 700.0021, and 699.8407 after aerial oxidation. Calculated molecular weight (MW) of day0 (immediately after dissolving) was 4199.0568 which is reduced linear peptide, while that of day7 (after aerial oxidation) was 4193.044 (decreased approximately 6 of MW) implied that formation of three intramolecular disulfide bridges.

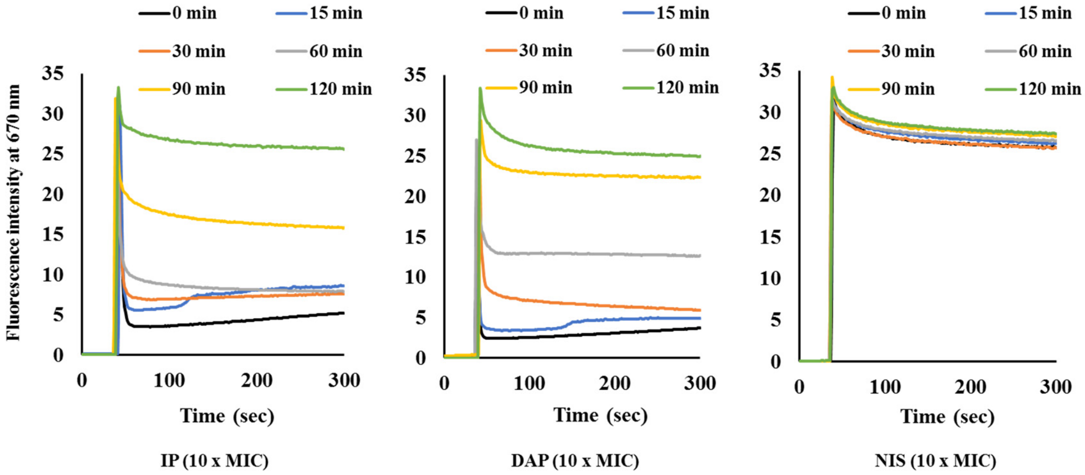

Supplementary Figure S2. Measurement of fluorescence during exposure to AMPs (IP, daptomycin, or nisin) using DiSC3(5) probe. Fluorescence intensity at 670 nm in excitation at 622 nm using fluorescence probe 3,3′-dipropylthiadicarbocyanine iodide (DiSC3(5)). Bacterial cells grown to exponential phase (OD at 660 of 0.5-0.6) were treated with 10×MIC of AMP such as IP (10 μg/mL), daptomycin (DAP, 20 μg/mL), and nisin (NIS, 2,560 μg/mL). After incubation for 0, 15, 30, 45, 60, 90, and 120 min, fluorescence intensity at 670 nm of cells were measured for 5 min with excitation at 622 nm. First, background data were collected for 30 sec before adding a DiSC3(5) probe (final concentration 1 μM). Then, the fluorescence intensity data were collected for an additional 4.5 min.

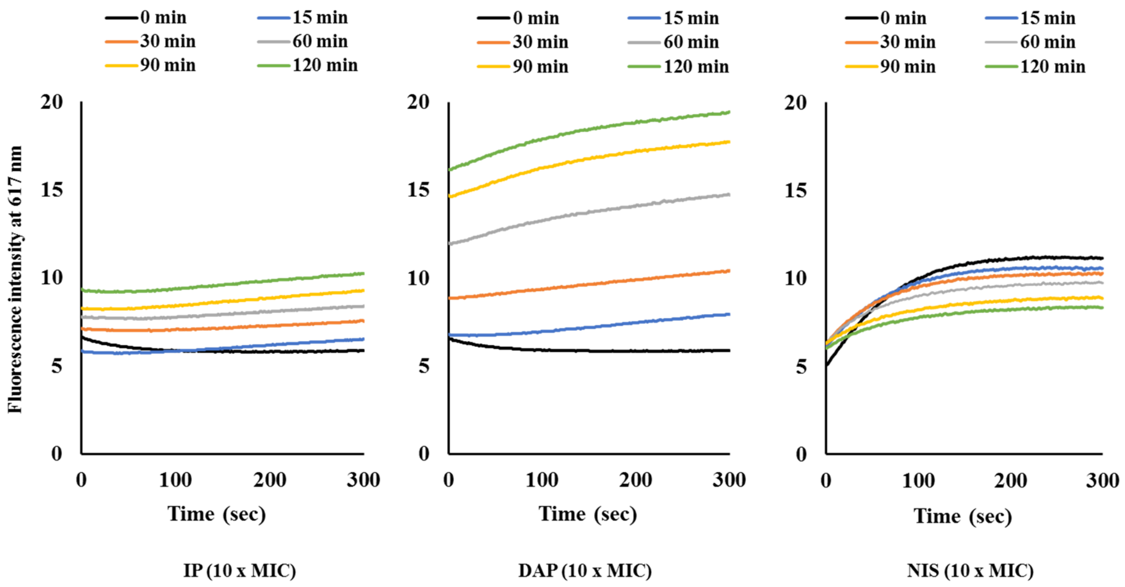

Supplementary Figure S3. Measurement of fluorescence during exposure to AMPs (IP, daptomycin, or nisin) using PI probe. Fluorescence intensity at 617 nm in excitation at 535 nm using fluorescence probe propidium iodide (PI). Bacterial cells grown to exponential phase (OD at 660 of 0.5-0.6) were treated with 10 x MIC of AMP such as IP (10 μg/mL), daptomycin (DAP, 20 μg/mL), and nisin (NIS, 2,560 μg/mL). After incubation for 0, 15, 30, 45, 60, 90, and 120 min, PI was added to a final concentration of 10 μM. Then, then fluorescence intensity at 617 nm of cells were measured for 5 min with excitation at 535 nm.
